# The relationship between financial difficulty and childhood symptoms of attention deficit/hyperactivity disorder: a UK longitudinal cohort study

**DOI:** 10.1007/s00127-017-1453-2

**Published:** 2017-11-09

**Authors:** Abigail Emma Russell, Tamsin Ford, Ginny Russell

**Affiliations:** University of Exeter Medical School, St Luke’s Campus, 2.05d South Cloisters, St Luke’s Campus, Exeter, EX1 2LU UK

**Keywords:** ADHD, Deprivation, Social environment, ALSPAC, Financial difficulty

## Abstract

**Purpose:**

Attention deficit/hyperactivity disorder (ADHD) is associated with socioeconomic status (SES), in that children who grow up in low SES families are at an increased risk of ADHD symptoms and diagnosis. The current study explores whether different levels of ADHD symptoms are associated with prior changes in the SES facet of financial difficulty.

**Methods:**

Using the Avon Longitudinal Study of Parents and Children (ALSPAC), we examined symptoms of ADHD measured by the Strengths and Difficulties Questionnaire (SDQ) hyperactivity subscale in relation to parent-reported changes in financial difficulty, grouped into four repeated measures at four time points across childhood; (*n* = 6416). A multilevel mixed-effects linear regression model with an unstructured covariance matrix was used to test whether different patterns of financial difficulty were associated with subsequent changes in ADHD symptoms.

**Results:**

Families who had no financial difficulty had children with a lower average ADHD symptom score than groups who experienced financial difficulty. Children whose families stayed in financial difficulty had higher mean ADHD symptom scores than all other groups (No difficulty mean SDQ hyperactivity 3.14, 95% CI 3.07, 3.21, In difficulty mean SDQ hyperactivity 3.39, 95% CI 3.28, 3.45, *p* < 0.001). Increasing or decreasing financial difficulty predicted mean symptom scores lower than those of the in difficulty group and higher than the no difficulty group.

**Conclusions:**

Our findings contribute to the building evidence that SES may influence the severity and/or impairment associated with the symptoms of ADHD, however the effects of SES are small and have limited clinical significance.

**Electronic supplementary material:**

The online version of this article (10.1007/s00127-017-1453-2) contains supplementary material, which is available to authorized users.

## Introduction

The aetiology of attention deficit/hyperactivity disorder (ADHD) is complex and multifaceted. Current theory suggests that multiple small common and rare genetic variants influence any individuals’ levels of inattention, hyperactivity and impulsivity, which when severely rise comprise the syndrome of ADHD [1]. Evidence around environmental factors that may influence vulnerability to ADHD often centres on prenatal exposures to toxins, such as those associated with smoking and alcohol consumption [2]. More recently, it has been accepted that social factors, such as socioeconomic status (SES), throughout the life-course may have a role in the aetiology of inattentive and impulsive behaviour that characterises ADHD [1, 3].

A diagnosis of ADHD is associated with an increased risk of negative outcomes for the individual across many domains including problems with social function and occupation, poor academic outcomes, driving and car accidents and increased use of services [4]. The prevalence of ADHD is estimated at 2–5% worldwide [5]. In spite of this, relatively little is known about its association with social and environmental factors early in life, such as SES [3]. In a recent study, we found that children whose mothers reported financial difficulty (FD) were over twice as likely to receive a research diagnosis of ADHD when the child was age seven [6]. FD can be understood and conceptualised as a measure of SES, in that it is likely to reflect availability and impact of economic resources or wealth [7]. The measure concerned asks directly about ability to afford basic necessities, such as food and housing, in a manner that considers the difficulty or burden this may cause the family.

Other studies have found associations between SES across childhood and mental health [8–11]. They have begun to unpick the impact of changing or persistent SES on a variety of mental health outcomes. One study found that the length of time a child spends in poverty has an increasingly detrimental impact on their mental health (specifically antisocial behaviour) [12]. The authors found that different outcomes may have different relationships with SES. More recent studies have also found that a clear difference in cognitive and socio-emotional development by SES was evident by age three and widened by age five [13]. With regard to stability of SES and impacts on mental health, Kiernan and Mensah found that 18% of children in persistently poor families between the age of 0–3 had behavioural problems compared with 4% of those who were not persistently poor [9]. In addition, Anselmi et al. found that not only did low income both at birth and at age 11 predict conduct problems at age 15; this also applied those who became poor between birth and 11 [8]. Decreasing SES throughout childhood may therefore result in an increase in externalising problems [8].

Some have suggested the ADHD–SES association is likely due to social selection: adolescents with ADHD are less likely to have good educational outcomes and this could determine low SES circumstances for them. As ADHD is highly heritable, the offspring of these individuals, genetically predisposed to ADHD, will be born into socioeconomically disadvantaged circumstances [14, 15]. Others argue that having a child with ADHD causes the parents’ SES to decrease due to disruption to ability to work [16]. A third alternative is that SES–ADHD associations are due to social causation: a mechanism by which SES exerts an influence on the aetiology or severity of ADHD. This is not mutually exclusive to the social selection theory [14, 15].

The current study aims to explore whether recent changes in FD are associated with different levels of ADHD symptoms following this change. If changing financial difficulties are associated with later changes in ADHD symptoms, it would suggest that factors associated with such socioeconomic disadvantage may play a causal role in aetiology of ADHD, rather than being due to social selection. Increasing family FD followed by higher levels of hyperactivity and inattention would suggest factors associated with SES are on the causal pathway.

We utilised data from the Avon Longitudinal Study of Parents and Children (ALSPAC) to examine symptoms of ADHD in children, grouped by change in FD between two measures, four times across childhood. This allowed us to address our question of interest: whether changes in FD are associated with subsequent differences in levels of ADHD symptoms.

## Methods

### Sample

ALSPAC is a longitudinal birth cohort in the UK that initially aimed to recruit all pregnant women living in the county of Avon with estimated delivery dates between 1st April 1991 and 31st December 1992 [17, 18]. 14,701 of these children were alive at 1 year of age. ALSPAC did not enrol triplet or quadruplet births in the cohort and in the case of twin pairs, one was included at random in the current sample. ALSPAC collected data on the mother and child from pregnancy and throughout the child’s lifespan through a series of questionnaires and clinical assessments. The initial study measures are therefore prior to the birth of the study child.

This study included all children who had at least partial data on the measures required to address our research questions, i.e. at least two consecutive FD measures and one ADHD symptom measure. The ALSPAC study website contains details of all the cohort data that is available through a fully searchable data dictionary [19]. Ethical approval for the study was obtained from the ALSPAC Ethics and Law Committee and the Local Research Ethics Committees, and the University of Exeter Medical School Research Ethics Committee.

### Measures

#### Exposure variable: financial difficulty change group

The financial difficulties measure was constructed of a series of five questions. The mother was asked to rate on a scale from zero to three how difficult it is currently to afford food, clothes, heating, rent/mortgage and other things considered essential for the child, with higher scores indicating more difficulty. We chose this as our exposure as it was the SES measure most highly predictive of ADHD in a previous study with the ALSPAC cohort [6], and because it was repeatedly measured five times between gestation and when the child was aged 12 (see Table 1). Twelve is the cut-off age for the manifestation of symptoms of ADHD in the DSM-5 diagnostic criteria.

**Table 1:** Measurement occasions for each repeated measure entered into multilevel model

Analysis	Child age at measurement occasion (months)			Period over which financial difficulty change calculated (months)	Time from second financial difficulties measurement to outcome (months)
	Financial difficulties 1	Financial difficulties 2	SDQ hyperactivity		
A	− 2	33	47	35	14
B	33	61	81	28	20
C	61	85	115	24	30
D	85	133	140	48	7

Each measurement occasion A-D comprises two financial difficulty measurements and a subsequent ADHD symptom outcome measure

Notes: Letters A–D indicate the four repeated measures for the study, each comprising two financial difficulty measures over which change is calculated, and the Strengths and Difficulties Questionnaire- hyperactivity subscale outcome (SDQ Hyperactivity)

For the main analysis, and because the majority of participants reported no FD, we dichotomised this measure into no FD (score of 0) vs any FD (score of 1 or more) at each time point that the measure was reported. Sensitivity analyses used thresholds of ≥ 5 and ≥ 10 (out of 15) to represent thresholds corresponding to those experiencing moderate and severe FD respectively.

#### Outcome variable: ADHD symptoms

Symptoms of ADHD were measured using the parent-report version of the hyperactivity subscale of the Strengths and Difficulties Questionnaire (SDQ) [20]. This scale asked about five symptoms of ADHD. Parents are asked to indicate whether these behaviours are “not true” (scored 0) “somewhat true” (scored 1) or “certainly true” (scored 2) of their child in the past 6 months. Scores are added for a total out of ten in the subscale, with higher scores indicating more symptoms. The SDQ is frequently used in clinical and research assessments of ADHD [20–22] and the scores correlate meaningfully with other validated ADHD symptom measures [22–24]. We utilised the parent-report version that mothers filled in about their child four times across childhood (Table 1).

#### Covariates

We included a range of covariates. These included child age at completion of the measures of financial difficulty and hyperactivity in months from the child’s birth date to the date the parent reported filling in the questionnaire. Child gender and birthweight (in grams) were also included, as were whether the child resided in a family with more than three biological children or not (large family size), and whether or not parents of the study child had experienced depression in the first 2 years of the child’s life based on a score of 13 or more on the Edinburgh Postnatal Depression Scale.

Covariates related to SES were also included:

- Housing tenure: mothers reported during pregnancy whether they lived in social or council housing, privately rented or owned/mortgaged a property.
- Parental employment. Maternal and paternal employment during pregnancy were categorised into unemployed, stay at home parent or in education, and employed.
- Weekly income, self-reported by mothers in £100 bands from £100 or less to £400 or more when the child was aged 33 months.
- Maternal education. Mothers reported during pregnancy on their highest educational attainment. This was categorised into not completing GCSE (the UK’s exams to mark the end of mandatory schooling), GCSE, or higher education than GCSE.
- Marital status during pregnancy. This was categorised as single, cohabiting or married.

Parity (number of times the mother had given birth prior to the birth of the study child) was also included as a covariate.

### Analysis

#### Defining financial difficulty change (FD change) groups

We defined four groups of FD change, each calculated from two consecutive FD measurements. Participants who were below the threshold for FD at both the first and second measurement occasion were classed as being in “No difficulty”. “Decreasing difficulty” participants were above the threshold for FD (i.e. in financial difficulty) at the first measurement occasion, and below it at the second (i.e. no longer in difficulty). “Increasing difficulty” participants were below the FD threshold at the first measurement occasion and above it at the subsequent occasion, and participants who were above the threshold and in FD at both measurement occasions were categorised as “In difficulty”. Each individual, therefore, had up to four data points across childhood for *FD change group* if they had complete data for all five FD measurement occasions.

#### Defining analysis time frames

**Fig. 1:**
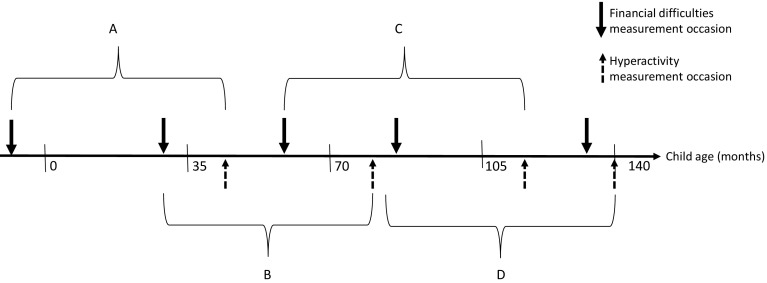
Measurement occasions and analysis groupings (not to scale) for the predictor: change in financial difficulties, and outcome: ADHD symptoms from the hyperactivity subscale of the Strengths and Difficulties Questionnaire. Note: Letters A–D indicate the four repeated measures for the study, each comprising two financial difficulty measures over which change is calculated, and the Strengths and Difficulties Questionnaire-hyperactivity subscale outcome

Each FD change period was analysed in relation to the nearest subsequent measurement of the outcome, SDQ hyperactivity in ALSPAC. These are outlined in Table 1 and Fig. 1. Due to the lack of standard intervals between measures in ALSPAC, each of the four FD change measures had a different *FD change* period and a different length of time to outcome.

#### Analysis

A multilevel mixed-effects linear regression model with an unstructured covariance matrix was used to test whether children in different FD change groups have different subsequent levels of ADHD symptoms using the “xtmixed” command in Stata 13. As we had repeated measures for each child in the study, this mixed model took these into account with random effects for repeated measures (level 1) within children (level 2), with the other variables being included as fixed effects. These fixed-effects coefficients are equivalent to and can be interpreted as standard regression coefficients.

To determine which covariates were significantly predictive of SDQ score across the four time points, in addition to FD change and child age at SDQ score measurement, we first ran the full model including all variables outlined above to determine which covariates were statistically significant (at the 0.05 threshold), re-ran the model with only the significant covariates and then introduced the other covariates individually; any further significant covariates were added to the model. We used likelihood ratio tests to determine whether these covariates improved the model fit alongside the Akaike information criterion (AIC) values.

We conducted a sensitivity analysis utilising two more stringent thresholds for FD of ≥ 5 and ≥ 10 (out of 15) to evaluate whether any findings are replicated or indeed are more pronounced using more stringent criteria. We then repeated the models using different FD change groups as the reference category to determine how each differed from the others. We used observed data only and did not impute missing data as those of low SES and with children who have higher scores on the SDQ are more likely to have missing data or drop-out from ALSPAC, and are thus not at missing at random [25].

## Results

### Descriptive

Available data for the sample varied by measurement occasion, however the mixed effects model included data from 6416 individuals (*n* observations = 19,574). This reflects the drop-out in ALSPAC and higher proportion of uncompleted measures as the children age, however the sample was still substantial. Descriptive statistics for the repeated measures model are described in Table 2. Mean SDQ hyperactivity scores decreased over the course of childhood.

**Table 2:** Descriptive statistics for study sample

	Repeated measures model (*n* obs = 19,574)			SDQ hyperactivity	
	Frequency (*n*)	%		Mean	SD
			Overall	2.26	2.33
FD change group (threshold ≥ 1): main analysis					
No difficulty	6537	33.4		2.77	2.19
Increasing difficulty	2067	10.56		3.35	2.28
Decreasing difficulty	2851	14.57		3.22	2.30
In difficulty	8119	41.48		3.66	2.39
FD change group (threshold ≥ 5)					
No difficulty	14,323	73.17		3.05	2.25
Increasing difficulty	1406	7.18		3.85	2.44
Decreasing difficulty	1866	9.53		3.55	2.23
In difficulty	1979	10.11		4.10	2.53
FD change group (threshold ≥ 10)					
No difficulty	21,956	91.28		3.22	2.31
Increasing difficulty	755	3.14		4.25	2.58
Decreasing difficulty	921	3.83		3.94	2.5
In difficulty	422	1.75		4.24	2.63
Covariates					
Estimated weekly income (£)					
< 100	939	4.80			
100–199	2844	14.53			
200–299	5736	29.3			
300–399	4668	23.85			
> 400	5387	27.52			
Housing tenure					
Council association	1653	8.44			
Private rented	950	4.85			
Owned or mortgage	16,971	86.7			
Marital status					
Single parent	1357	6.93			
Cohabiting	1712	8.75			
Married	16,505	84.32			
Maternal education level					
< GCSE	3873	19.79			
GCSE	6952	35.52			
> GCSE	8749	44.7			
Paternal employment					
Unemployed	1070	5.47			
Stay at home, retired, in education	387	1.98			
Employed	18,117	92.56			
Maternal employment					
Unemployed	365	2.16			
Stay at home, retired, in education	7582	44.89			
Employed	8942	52.95			
Parity (number of prior pregnancies)					
0	8874	45.34			
1	7303	37.31			
2	2551	13.03			
3	666	3.40			
4	141	0.72			
5	39	0.20			
Large family size (> 3 biological children in family)					
Yes	835	4.27			
No	18,739	95.73			
Gender					
Male	10,002	51.10			
Female	9572	48.90			
Maternal depression between child age 0–3					
Yes	2131	10.89			
No	17,443	89.11			
Paternal depression between child age 0–2					
Yes	358	2.74			
No	12,691	97.26			

	mean (SD)				
Birthweight (grams)	3459 (507)				
Child age at FD measurement 1 (months)	38.35 (32.56)				
Child age at FD measurement 2 (months)	71.73 (35.78)				
Age at SDQ measurement	89.75 (34.97)				

Notes: *n* for each variable differ due to data available. FD: financial difficulty, SDQ: strengths and difficulties questionnaire. Threshold ≥ 1 refers to main analysis, any financial difficulty vs none used to determine grouping. Threshold ≥ 5 and ≥ 10 represent more severe cutoffs on the financial difficulty scale (/15), thus represent analyses with moderate and severe cutoffs where an individual is considered to be in financial difficulty

### Primary analysis

#### Differences in ADHD symptoms by financial difficulties change group

The multilevel mixed-effects linear regression model showed the best fit when including a large number of the covariates. Only age at ADHD symptom report, maternal employment and paternal depression did not significantly contribute to the model fit and so were not included in the final model.

**Fig. 2:**
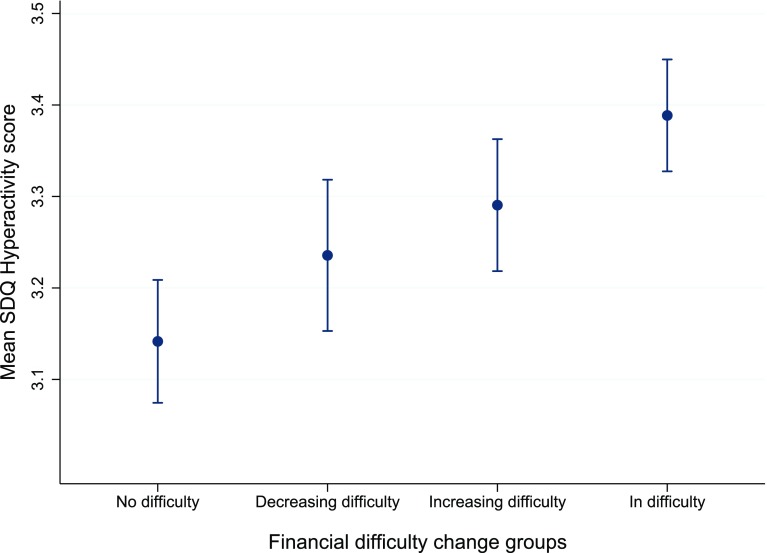
Marginal mean Strengths and Difficulties Questionnaire Hyperactivity values (95% CI) for multilevel model exploring association between financial difficulty change and ADHD symptoms. Notes: *Y* axis of graph starts at three for illustrative purposes only. *SDQ* Strengths and Difficulties Questionnaire

There was a significant association between recent FD and subsequent ADHD symptoms, with those in the In difficulty group having significantly higher ADHD symptom scores than all other FD change groups (No difficulty mean SDQ hyperactivity 3.14, 95% CI 3.07, 3.21, In difficulty mean SDQ hyperactivity 3.39, 95% CI 3.28, 3.45, *p* < 0.001: see Table 3 for full model marginal means and 95% confidence intervals and supplementary information Table 1 for coefficients and standard errors). Those in the In difficulty group had a mean SDQ hyperactivity score 0.25 SDQ points higher than the No difficulty group. Marginal mean SDQ scores and their standard errors are graphically represented in Fig. 2.

**Table 3:** Results from multilevel mixed-effects linear regression model exploring association between financial difficulty change and subsequent ADHD symptoms measured by the Strengths and Difficulties Questionnaire Hyperactivity subscale

Predictor	Threshold ≥ 1	*p*	Threshold ≥ 5	*p*	Threshold ≥ 10	*p*
	Mean SDQ hyperactivity score (95% CI)		Mean SDQ hyperactivity score (95% CI)		Mean SDQ hyperactivity score (95% CI)	
Financial difficulty change group		< 0.001		< 0.001		< 0.001
No difficulty (reference group)	3.14 (3.07, 3.21)		3.22 (3.17, 3.28)		3.26 (3.21, 3.31)	
Increasing difficulty	3.24 (3.15, 3.32)		3.36 (3.26, 3.47)		3.47 (3.31, 3.62)	
Decreasing difficulty	3.29 (3.22, 3.36)		3.36 (3.27, 3.45)		3.41 (3.28, 3.55)	
In difficulty	3.39 (3.28, 3.45)		3.51 (3.40, 3.62)		3.62 (3.39, 3.84)	

Covariates	Average marginal effect (95% CI)		Average marginal effect (95% CI)		Average marginal effect (95% CI)	
Estimated weekly income (£)						
< 100	Reference	0.02	Reference	0.02	Reference	0.003
100–199	-0.02 (− 0.25, 0.21)		0.01 (− 0.22, 0.52)		− 0.004 (− 0.24, 0.23)	
200–299	− 0.21 (− 0.47, 0.23)		− 0.15 (− 0.39, 0.08)		− 0.20 (− 0.43, 0.04)	
300–399	− 0.28 (− 0.52, − 0.04)		− 0.24 (− 0.48, 0.01)		− 0.29 (− 0.53, − 0.05)	
> 400	− 0.25 (− 0.50, − 0.01)		− 0.24 (− 0.48, 0.01)		− 0.30 (− 0.55, − 0.06)	
Housing tenure						
Council association	Reference	0.07	Reference	0.06	Reference	0.05
Private rented	− 0.02 (− 0.28, 0.24)		− 0.03 (− 0.28, 0.23)		− 0.02 (− 0.28, 0.24)	
Owned or mortgage	− 0.19 (− 0.37, − 0.01)		− 0.19 (− 0.38, − 0.01)		− 0.20 (− 0.38, − 0.01)	
Marital status						
Single parent	Reference	0.10	Reference	0.10	Reference	0.08
Cohabiting	− 0.21 (− 0.04, 0.01)		− 0.21 (− 0.44, 0.02)		− 0.21 (− 0.44, 0.02)	
Married	− 0.20 (− 0.38, − 0.01)		− 0.20 (− 0.39, − 0.01)		− 0.21 (− 0.40, − 0.02)	
Maternal education level						
< GCSE	Reference	< 0.001	Reference	< 0.001	Reference	< 0.001
GCSE	− 0.13 (− 0.26, 0.00)		− 0.12 (− 0.25, 0.01)		− 0.12 (− 0.25, 0.01)	
> GCSE	− 0.57 (− 0.71, − 0.44)		− 0.57 (− 0.70, − 0.43)		− 0.21 − (0.71, − 0.43)	
Paternal employment						
Unemployed	Reference	0.04	Reference	0.04	Reference	0.04
Stay at home, retired, in education	− 0.47 (− 0.84, − 0.10)		− 0.47 (− 0.84, − 0.09)		− 0.47 (− 0.85, − 0.10)	
Employed	− 0.06 (− 0.27, 0.14)		− 0.05 (− 0.26, 0.16)		− 0.06 (− 0.26, 0.15)	
Parity (number of prior pregnancies)						
0	Reference	0.06	Reference	0.08	Reference	0.08
1	0.12 (0.02, 0.23)		0.12 (0.01, 0.22)		0.12 (0.01, 0.22)	
2	− 0.06 (− 0.21, 0.09)		− 0.06 (− 0.21, 0.09)		− 0.05 (− 0.20, 0.10)	
3	0.25 (− 0.11, 0.60)		0.23 (− 0.12, 0.59)		0.24 (− 0.12, 0.59)	
4	0.02 (− 0.59, 0.63)		0.01 (− 0.60, 0.62)		0.02 (− 0.59, 0.63)	
5	− 0.32 (− 1.33, 0.70)		− 0.30 (− 1.32, 0.71)		− 0.29 (− 1.31, 0.73)	
Large family size (> 3 biological children in family)	− 0.42 (− 0.75, − 0.08)	0.02	− 0.42 (− 0.76, − 0.08)	0.02	− 0.41 (− 0.75, − 0.07)	0.02
Male gender	0.77 (0.67, 0.86)	< 0.001	0.77 (0.68, 0.86)	< 0.001	0.77 (0.68, 0.86)	< 0.001
Maternal depression present between child age 0–2	0.64 (0.50, 0.79)	< 0.001	0.64 (0.49, 0.78)	< 0.001	0.65 (0.50, 0.79)	< 0.001
Birthweight (g)	− 0.0002 (− 0.0003, − 0.0001)	< 0.001	− 0.0002 (− 0.0003, − 0.0001)	< 0.001	− 0.0002 (− 0.0003, − 0.0001)	< 0.001
Age at financial difficulty measurement 1 (months)	− 0.23 (− 0.03, − 0.02)	< 0.001	− 0.02 (− 0.03, − 0.02)	< 0.001	− 0.02 (− 0.03, − 0.02)	< 0.001
Age at financial difficulty measurement 2 (months)	0.01 (0.005, 0.011)	< 0.001	0.01 (0.006, 0.010)	< 0.001	0.01 (0.005, 0.010)	< 0.001

* N* obs 19,574.* N* individuals 6,416

#### Changing financial difficulty groups do not differ from each other

The Increasing and Decreasing difficulty groups were associated with higher subsequent ADHD symptom scores relative to those in the No difficulty group: those in the Increasing group had mean SDQ scores of 3.24 (95% CI 3.15, 3.32) and those in the Decreasing group had a mean score of 3.29 SDQ points (95% CI 3.22, 3.36). The changing FD groups were significantly different from the No difficulty group and the In difficulty group (*p* < 0.05) but not from each other (see Fig. 2).

The average SDQ hyperactivity score for males (3.65, 95% CI 3.59, 3.72) was 0.77 points higher than for females (2.88, 95% CI 2.82, 2.95, *p* < 0.001). Post hoc analyses found no significant interaction between gender and FD change group. The age of the child when FD were measured had a significant influence on SDQ hyperactivity score (*p* < 0.001 for both measurement time points). Higher income and maternal education, smaller family size and absence of maternal depression were associated with lower hyperactivity scores. Lower birthweight was associated with higher hyperactivity scores. Although parity, marital status and housing tenure contributed to model fit, they were not significantly associated with the outcome.

#### Using more stringent thresholds for financial difficulty does not alter the findings

The trend for ADHD symptom scores was found for the more severe thresholds for “financial difficulty”, with in almost all cases there being the lowest coefficient for the No difficulty group, the Increasing and Decreasing FD groups not differing from each other, and the In difficulty group having a significantly higher coefficient than all other groups (see Table 3). The mean difference between the changing FD groups and the reference group (No difficulty) was lower than the mean difference between the reference group and the In difficulty group. Increasing the stringency of the threshold supported these results: the average SDQ hyperactivity scores for each FD change group were higher as FD was defined more stringently. For example, the In difficulty group mean symptom scores were 3.39 (95% CI 3.28, 3.45) for threshold ≥ 1, the primary analysis, 3.51 (95% CI 3.40, 3.62) for threshold ≥ 5, moderate threshold for considering an individual is in FD, and 3.62 (95% CI 3.39, 3.84) for threshold ≥ 10 (severe threshold for FD).

## Discussion

### Different experience of financial difficulties is associated with different levels of ADHD symptoms

We evaluated a change in FD over four time points during childhood in relation to subsequent ADHD symptoms as measured by the parent-report SDQ hyperactivity subscale. This allowed us to explore how recent changes in family financial difficulty may be associated with subsequent variation in children’s ADHD symptoms. In a mixed effects model combining all measures, we found that those who reported no FD at two consecutive time points had a lower average symptom score than all other groups: implying that those of higher SES would have lower levels of ADHD symptoms. We also found that those children who were in FD across two time points had a higher mean SDQ score than all other groups. The two groups defined by change in FD had intermediate mean ADHD symptom scores that differed significantly from both the stable groups. Of interest, there was a negligible difference between the coefficient sizes of the two changing FD groups.

### Any experience of financial difficulty is associated with increased ADHD symptoms

The implications of our findings are that any experience of FD is associated with higher subsequent hyperactivity scores of around 0.1–0.3 SDQ points relative to those in no difficulty. This value increased with more stringent thresholds being used to define being in “financial difficulty”, with those analysed using the severe threshold for FD having SDQ scores around 0.2–0.4 points higher relative to those in no difficulty. This is suggestive of a trend where those who are the most disadvantaged have larger associations between FD and ADHD symptoms. Our results also suggest that the experience of any financial difficulty at any time is associated with higher levels of subsequent ADHD symptoms. This demonstrates that regardless of the mechanisms by which this association occurs, there is a small but significant longitudinal relationship between recent FD change and symptoms of ADHD.

Our findings are of aetiological interest but have limited clinical significance. The hyperactivity scale is often used as part of a multi-dimensional assessment of ADHD [20–22], and correlates with other measures of ADHD symptoms [22, 23]. The parent-report version of the SDQ has a specificity of 92% and sensitivity of 74% for a diagnosis of ADHD, although it should be noted that these figures were calculated using the impact supplement of this questionnaire, which data were not collected in ALSPAC [20]. Higher scores on the SDQ are related to an increased risk of meeting diagnostic criteria for ADHD, especially for those already close to thresholds.

### Our findings in the context of social selection

To draw inferences from our findings in line with theories of social selection, we need to consider what level of ADHD symptoms one would expect to find if the relationship between SES and ADHD was entirely due to fixed genetic effects. Symptoms of ADHD would be expected to be stable regardless of changes in SES, so those born into high SES families at birth would have lower mean ADHD symptoms than those born into lower SES families. A change in SES would not exert an effect on ADHD symptoms. We did not find this, instead we found those in the changing FD groups had ADHD symptom levels that lay between those of the stable SES children. There are three potential explanations:

First, symptoms of ADHD are temporally associated with FD, but due to constraints of measurement occasions the pattern of change was not observed. Second, the results could illustrate a ‘dose–response’ relationship where any experience of FD leads to an increase in ADHD symptoms, with higher levels of exposure having an additive effect on the association with symptoms. Third, there may be a difference in genetic susceptibility to ADHD symptoms between those of low, changing and high FD: those in constant FD having the highest genetic risk for ADHD; changing FD families having a moderate genetic risk and some ADHD traits that lead to them being unable to provide a stable environment for their child, whose symptom levels reflect this. Those constantly not in FD would, therefore, represent those with the least genetic risk, and in each case genetic risk would be associated both with ADHD traits and FD.

Overall our study did not provide conclusive evidence to discount selection effects, but greater socioeconomic disadvantage was shown to be associated with more ADHD symptoms and no reported financial difficulty was associated with lower levels of ADHD symptoms. The mechanisms of this effect can only be disentangled further with studies that account for parental ADHD traits and have sufficient data to closely track changes in all the variables of interest. Although the mechanisms of how changing SES may impact on symptoms of ADHD are as yet unclear, theory suggests that psychosocial stressors may impact on the family environment and parenting behaviours and lead to increased ADHD behaviours. Others posit that material possessions related to financial status may also be mechanisms through which this association may operate, for example, by not being able to afford educational and stimulating home-learning materials [26, 27].

### SES as a complex concept that may exert effects through a range of mechanisms

This study controlled for a variety of potential confounders including other baseline indicators of SES, such as income and education, or those commonly associated with SES, such as birthweight [7]. ALSPAC has inherent limitations in that data collected do not always meet methodological ideals, as such we used a measure of parental depression as proxy for parental psychopathology because no measures more closely related to parental ADHD were available. Our aim was to identify the conceptual relationship between FD change and ADHD symptoms, and we found that this association was robust even adjusting for more material measures of SES. This has implications for understanding the course and exacerbation of ADHD symptoms.

Our findings, if replicated, have implications for policy, health and special educational service delivery as we found that experiencing financial difficulty or stress is at the very least associated with a small increased risk of ADHD symptoms in children. ADHD symptoms have been shown to be associated with substantially lower academic achievement in the ALSPAC cohort [28]. The broader SES–ADHD association could translate to poorer health and educational outcomes for children growing up in disadvantaged socioeconomic circumstances, which is increasing during these austere times. The use of the subjective measure of financial difficulty as a measure of SES reflects whether the mother feels that she struggles to afford food, housing, heating, clothing and necessities for the child: all acknowledged to be essential for basic living standards. The measure has no objective standard; however, at all times the majority of participants reported that they experienced no financial difficulty at all, as may be expected based on the ALSPAC sample demographics. This suggests that those who report difficulty are likely to experience a real difference in financial stress [18, 25]. There are alternative hypotheses that may further explain the temporal association between SES and symptoms of ADHD, these are investigated in depth in a separate study (Russell et al., in preparation) and find that cumulative exposure to financial difficulty in early childhood (up to age seven) is also associated with symptoms of ADHD.

### Strengths and limitations

Whilst we did find evidence that different experiences of FD are associated with different levels of ADHD symptoms, this is somewhat difficult to interpret as both the groups representing changing FD (rather than stable FD) had similar coefficient values. This may be due to the limited range of measurement occasions: depending on when a family’s circumstances change and the amount of time before there is a change in the child’s behaviour, children will have different patterns of change. One limitation of the study was that all measures were reported by one individual, the mother. Utilising teacher-reported ADHD symptoms may address this; however these were only available on two occasions across childhood in ALSPAC.

The longitudinal design of the study was a strength, and repeated measures allowed us to draw conclusions across childhood rather than only at individual time points. In addition, using a variety of thresholds to define FD allowed us to test whether the association was robust when more stringent thresholds for defining low SES were used, and the results showed that if anything those that are more disadvantaged have higher symptom levels. Finally, we found that including the age of the child when FD was measured in the model had effects in different directions at the earlier and later time point. This finding was intriguing and should be further explored in other studies.

One study recently reported that poverty longitudinally predicted increased externalising behaviour problems, including hyperactivity, across early to middle childhood, supporting our findings [11]. Our findings also concur with those discussed earlier [8, 9, 12, 13], but have not been able to unpick how changing SES may affect symptoms of ADHD. Another study found associations between externalising problems and family income in the same direction as our study found [29]. In addition, the authors found that children living in chronically poor families benefitted most from an increase in income, implying that increasing SES may ameliorate externalising symptoms.

### Future directions

This study indicates that increasing financial difficulty has a negative impact on symptoms of ADHD, and that higher SES is associated with lower levels of ADHD symptoms. However, as the mechanisms by which this association operates have not been elicited, further research needs to determine mediators of the aetiological mechanisms before consideration of implications for policy and practice, especially with studies beginning to emerge that demonstrate that experiences of severe socio-emotional deprivation may be associated with persistent ADHD [10]. If this is the case for early experiences of socioeconomic deprivation as some posit, policy changes now could reduce the burden of ADHD in the future [6, 30]. Observational studies should explore whether socioeconomic changes in a family lead to changes in family environment or reduce biological markers of stress. These should be complemented by studying the relation between these social and environmental factors and symptoms of ADHD, of which some research already exists [31].

Our findings could not provide conclusive evidence around whether FD changes are in addition to or interact with the complex genetic heritability of ADHD. Recent research exploring interaction between genotypes and environmental exposures is beginning to allow us to tease apart the interrelation between these factors [32]. It may be that a combination of genetic predisposition and social/environmental adversity interact to exacerbate or ameliorate ADHD symptoms in a differential manner across childhood. Future studies with more detailed data on SES and more frequent measures could address whether children in families that have changing SES do show linear patterns of improvement or exacerbation of symptoms, and the extent to which symptoms can fluctuate.

## Conclusion

This study demonstrated an association between financial difficulty and childhood symptoms of ADHD that was robust to changes in the threshold used to define FD and timing of the measurements. Our findings add to the building evidence that SES may influence the severity and/or impairment associated with the symptoms of ADHD.

## Electronic supplementary material

Below is the link to the electronic supplementary material.

Supplementary material 1 (DOCX 26 KB)

## Abbreviations

ADHD: Attention deficit/hyperactivity disorder
FD: Financial difficulty
SES: Socioeconomic status
