# Supplementary material for: The relationship between financial difficulty and childhood symptoms of attention deficit/hyperactivity disorder: a UK longitudinal cohort study
Source: Soc Psychiatry Psychiatr Epidemiol. 2017 Nov 9;53(1):33–44. doi: 10.1007/s00127-017-1453-2 (PMC5846873; doi:10.1007/s00127-017-1453-2)
Supplement: Supplementary file 1 — Supplementary material 1 (DOCX 26 KB) [file 127_2017_1453_MOESM1_ESM.docx]

# Supplementary Information

## The relationship between financial difficulty and childhood symptoms of attention deficit/hyperactivity disorder: a UK longitudinal cohort study: Social Psychiatry and Psychiatric Epidemiology

Abigail Emma Russell^1^, Tamsin Ford and Ginny Russell

^1^University of Exeter Medical School, St Luke’s Campus, Exeter. EX1 2LU [a.e.russell@exeter.ac.uk](mailto:a.e.russell@exeter.ac.uk)

### Supplementary Table 1: Results from multilevel mixed-effects linear regression model exploring association between financial difficulty change and SDQ Hyperactivity: coefficients and standard errors (N obs=19,574)

|  |  | **Threshold ≥1** |  |  | **Threshold ≥5** |  |  | **Threshold ≥10** |  |
| --- | --- | --- | --- | --- | --- | --- | --- | --- | --- |
| **Predictor** |  | **Coefficient (SE)** | **p** |  | **Coefficient (SE)** | **p** |  | **Coefficient (SE)** | **p** |
|  |  |  |  |  |  |  |  |  |  |
| **Financial Difficulties Change Group** |  |  |  |  |  |  |  |  |  |
| No difficulty |  | *reference group* | <0.001 |  | *reference group* | <0.001 |  | *reference group* | 0.002 |
| Increasing difficulty |  | 0.09 (0.05) |  |  | 0.13 (0.05) |  |  | 0.21 (0.08) |  |
| Decreasing difficulty |  | 0.15 (0.04) |  |  | 0.13 (0.05) |  |  | 0.15 (0.07) |  |
| In difficulty |  | 0.25 (0.04) |  |  | 0.29 (0.06) |  |  | 0.36 (0.11) |  |
|  |  |  |  |  |  |  |  |  |  |
| ***Covariates:*** |  |  |  |  |  |  |  |  |  |
| **Estimated weekly income (£)** |  |  |  |  |  |  |  |  |  |
| <100 |  | *reference* | 0.02 |  | *reference* | 0.02 |  | *reference* | 0.003 |
| 100 - 199 |  | -0.02 (0.12) |  |  | 0.01 (0.12) |  |  | -0.004 ('-0.12) |  |
| 200 - 299 |  | -0.21 (0.12) |  |  | -0.15 (0.12) |  |  | -0.20 (0.12) |  |
| 300 - 399 |  | -0.28 (0.12) |  |  | -0.24 (0.12) |  |  | -0.29 (0.12) |  |
| >400 |  | -0.25 (0.12) |  |  | -0.24 (0.13) |  |  | -0.30 (0.12) |  |
| **Housing tenure** |  |  |  |  |  |  |  |  |  |
| council association |  | *reference* | 0.07 |  | *reference* | 0.06 |  | *reference* | 0.05 |
| private rented |  | -0.02 (0.13) |  |  | -0.03 (0.13) |  |  | -0.02 (0.13) |  |
| owned or mortgage |  | -0.19 (0.09) |  |  | -0.19 (0.09) |  |  | -0.20 (0.09) |  |
| **Marital status** |  |  |  |  |  |  |  |  |  |
| single parent |  | *reference* | 0.10 |  | *reference* | 0.10 |  | *reference* | 0.08 |
| cohabiting |  | -0.21 (0.12) |  |  | -0.21 (0.12) |  |  | -0.21 (0.12) |  |
| married |  | -0.20 (0.10) |  |  | -0.20 (0.10) |  |  | -0.21 (0.10) |  |
| **Maternal education level** |  |  |  |  |  |  |  |  |  |
| <GCSE |  | *reference* | <0.001 |  | *reference* | <0.001 |  | *reference* | <0.001 |
| GCSE |  | -0.13 (0.07) |  |  | -0.12 (0.07) |  |  | -0.12 (0.07) |  |
| >GCSE |  | -0.57 (0.07) |  |  | -0.57 (0.07) |  |  | -0.21 (0.10) |  |
| **Paternal employment** |  |  |  |  |  |  |  |  |  |
| unemployed |  | *reference* | 0.04 |  | *reference* | 0.04 |  | *reference* | 0.04 |
| stay at home, retired, in education |  | -0.47 (0.19) |  |  | -0.47 (0.19) |  |  | -0.47 (0.19) |  |
| employed |  | -0.06 (0.10) |  |  | -0.05 (0.10) |  |  | -0.06 (0.11) |  |
| **Parity (number of prior pregnancies)** | |  |  |  |  |  |  |  |  |
| 0 |  | *reference* | 0.06 |  | *reference* | 0.08 |  | *reference* | 0.08 |
| 1 |  | 0.12 (0.45) |  |  | 0.12 (0.05) |  |  | 0.12 (0.05) |  |
| 2 |  | -0.06 (0.08) |  |  | -0.06 (0.08) |  |  | -0.05 (0.08) |  |
| 3 |  | 0.25 (0.18) |  |  | 0.23 (0.18) |  |  | 0.24 (0.18) |  |
| 4 |  | 0.02 (0.31) |  |  | 0.01 (0.31) |  |  | 0.021 (0.31) |  |
| 5 |  | -0.32 (0.52) |  |  | -0.30 (0.52) |  |  | -0.29 (0.52) |  |
|  |  |  |  |  |  |  |  |  |  |
| **Large family size (>3 biological children in family)** | | -0.42 (0.17) | 0.02 |  | -0.42 (0.17) | 0.02 |  | -0.41 (0.17) | 0.02 |
| **Male gender** |  | 0.77 (0.04) | <0.001 |  | 0.77 (0.05) | <0.001 |  | 0.77 (0.05) | <0.001 |
| **Maternal depression present between child age 0-2** | | 0.64 (0.08) | <0.001 |  | 0.64 (0.08) | <0.001 |  | 0.65 (0.08) | <0.001 |
|  |  |  |  |  |  |  |  |  |  |
| **Birthweight (grams)** |  | -0.0002 (0.00) | <0.001 |  | -0.0002 (0.00) | <0.001 |  | -0.0002 ('0.00) | <0.001 |
| **Age at FD measurement 1 (months)** |  | -0.23 (0.001) | <0.001 |  | -0.02 (0.001) | <0.001 |  | -0.02 (0.001) | <0.001 |
| **Age at FD measurement 2 (months)** |  | 0.01 (0.001) | <0.001 |  | 0.01 (0.001) | <0.001 |  | 0.01 (0.001) | <0.001 |

^In all cases the No difficulty trajectory coefficient is significantly lower than for all other groups (p<0.001), the In difficulty trajectory coefficient is significantly higher from all groups apart from for the highest threshold ( ≥10) and the two change groups (increasing and decreasing) do not differ significantly from each other and sit in between the In and No difficulty values. N=6,416. Thresholds refer to the cutoff for calculating change in financial difficulty on a scale from 0 to 15. FD: financial difficulty, SE: standard error^
